# Control of spontaneous charging of sliding water drops by plasma-surface treatment

**DOI:** 10.1038/s41598-024-60595-5

**Published:** 2024-05-09

**Authors:** Fahimeh Darvish, Sajjad Shumaly, Xiaomei Li, Yun Dong, Diego Diaz, Mohammadreza Khani, Doris Vollmer, Hans-Jürgen Butt

**Affiliations:** 1 Max Planck Institute for Polymer Research (MPI-P), Ackermannweg 10, 55128 Mainz, Germany; 2 Laser and Plasma Research Institute, Shahid Beheshti University, G.C., Evin, Tehran, 1983963113 Iran

**Keywords:** Water contact electrification, Low-pressure plasma treatment, Plasma-sheath, Adaptation, Surface charge neutralization, Wetting, Energy harvesting

## Abstract

Slide electrification is the spontaneous separation of electric charges at the rear of water drops sliding over solid surfaces. This study delves into how surfaces treated with a low-pressure plasma impact water slide electrification. Ar, O_2_, and N_2_ plasma treatment reduced the drop charge and contact angles on glass, quartz, and SU-8 coated with 1H,1H,2H,2H-perfluoroctyltrichlorosilane (PFOTS), and polystyrene. Conversely, 64% higher drop charge was achieved using electrode-facing treatment in plasma chamber. Based on the zeta potential, Kelvin potential, and XPS measurements, the plasma effects were attributed to alterations of the topmost layer's chemistry, such as oxidation and etching, and superficially charge deposition. The surface top layer charges were less negative after electrode-facing and more negative after bulk plasma treatment. As a result, the zeta potential was less negative after electrode-facing and more negative after bulk plasma treatment. Although the fluorinated layer was applied after plasma activation, we observed a discernible impact of plasma-glass treatment on drop charging. Plasma surface modification offers a means to adjust drop charges: electrode-facing treatment of the fluorinated layer leads to an enhanced drop charge, while plasma treatment on the substrate prior to fluorination diminishes drop charges, all without affecting contact angles or surface roughness.

## Introduction

Water drops moving down inclined solid surfaces spontaneously acquire charges and deposit counter charges onto the solid surface^1^. This spontaneous charge separation by sliding drops has been called “slide electrification”^2^. Motivated by fundamental questions^3^, slide electrification has been studied on many types of surfaces ^1,4^. In general, spontaneous charging between water and solids can occur when a drop slides on hydrophobic surfaces^5^, bounces off surfaces ^6,7^, is ejected from nozzles ^8^, or is pipetted from hydrophobic capillaries ^9^. Studies reveal that the drop charge depends on slide length^2^, dripping flow rate^10^, surface chemistry ^11,12^, pH value^13^, and salt concentration^4^. To explain the charge separation of sliding drops, it has been proposed that a part of the surface charge forming the electric double layer in liquid remains at the solid surface behind the receding contact line^5,14–16^. After transferring ions to solids, the environment neutralizes the ions left on the surface. It may happen due to ion migration/desorption and electron flow ^5,17^.

However, the effect of the solid surface on slide electrification is poorly understood. For example, it is unclear if and how plasma treatment of a hydrophobic layer or plasma treatment of the substrate prior to hydrophobization influences drop charging. Plasma is known to modify the surface chemistry and surface charges. Our experiments had two goals: First, we needed to create methods to manipulate drop charges in order to increase or decrease the charge of sliding drops by plasma treatment. Secondly, we examined how plasma-surface treatment influences charge separation in sliding drops. Plasma treatment is involved in many methods of modifying a solid surfaces, both chemically and physically. For example, when coating glass or silicon oxide with silanes, the substrate is often first activated by plasma treatment. Polymer surfaces^18^ are frequently plasma-treated to tune the hydrophobicity^19,20^, modifying surface charge^21^, fuctionalization^22^, radicalization and grafting^23–25^, crosslinking^23^, and etching^26,27^. Plasma is a chemical-free, ultrafast, low-cost, eco-friendly method for controlling surface chemistry, which are key factors in applications such as cell biology, sensors, microfluidics, plasma actuators for anti-/de-icing application^28–31^, etc. For example, the performance of plasma actuators in wet conditions is influenced by the presence of drops on its surface^32^. Designing actuators that make their substrates hydrophobic would be beneficial for anti-/de-icing, allowing plasma actuators to retain more thrust when exposed to water drops.

In general, plasma is an ionized gas composed of positive and negative ions, electrons, photons, and neutral species. These species are distributed in the bulk of the plasma chamber in quasi-neutral (the same density of electrons and ions) and thin non-neutral regions. In low-pressure plasma, there is a region, a few mm thick (e.g., DC glow discharge), enriched in positive space charge, close to the wall, called the sheath^33,34^. “The formation of sheaths is a consequence of the higher mobility of electrons, which can leave the plasma more easily than ions”^35^. The sheath width depends on plasma parameters, e.g., feed gas type and pressure, frequency, wall bias^36^ and so on. A passive sheath (i.e. one without any rf field) forms along the edge of the plasma, acting as a barrier between the quasi-neutral plasma and the confining surface (a cathode, or occasionally an anode). In the absence of any net current, the width of this layer is a few Debye lengths. However, the introduction of high rf voltages causes a notable increase in the sheath's width and its dc voltage^37^.

The sheath region is commonly used for plasma etching applications, like microelectronic applications, as in normal and Deep Reactive Ion Etching (DRIE/RIE)^38^. To tune the plasma effects and gain deeper insights into the mechanisms of plasma-assisted surface charge and chemistry, samples in one experiment were faced towards the bulk plasma. In another experiment, samples faced the power electrodes of the plasma chamber to benefit from the sheath's positive space charge. “Space-charge sheaths are inherent to bounded plasmas because of the significant difference in the electron and thermal velocities”^37^.

**Figure 1:**
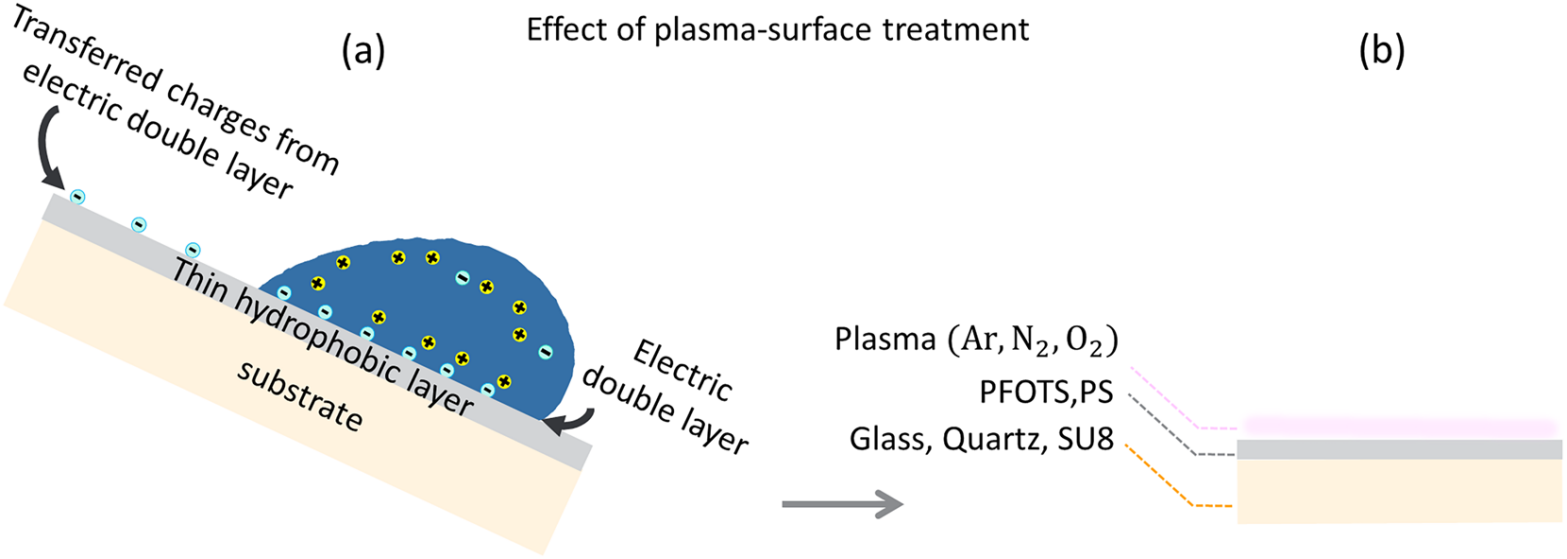
(**a**) Schematic of a water drop spontaneously depositing charges along its track on a hydrophobic surface. (**b**) Hydrophobic samples after plasma treatment.

In slide electrification, hydrophobic materials were used, such as polytetrafluoroethylene (PTFE)^13,39–41^, polydimethylsiloxane (PDMS)^42,43^, nylon^4^, and polyethylene^4^. On such hydrophobic surfaces, sliding drops leave negative charges on surface and acquire positive charges (Fig. 1a). Various hydrophobic microstructures have been fabricated to gain a high electric charge from drops^42^. Plasma treatment has also been utilized to enhance the output efficiency of triboelectric nanogenerators (TENG) through the physical and chemical modification of the surface, achieved by altering the surface charge, roughness, wettability, and chemistry^44–47^.

Plasma treatment is known as a technique used to change surface chemistry^48,49^ faster than applying chemical methods. For instance, partial defluorination (CA ≈ 112°–106°) of a fluorosilane layer was achieved after 10 min of oxidation with a piranha solution^50^. Fluorosilane and different hydrophobic materials became hydrophilic and superhydrophilic (CA < 10°) using plasma treatment in a few ten seconds ^51,52^. In addition, plasma can generate surface charges^53^. In conductive materials like indium gallium zinc oxides, plasma influences the overall charge balance in the material. For example, O_2_ and N_2_ plasma treatment reduces carrier concentration, while Ar treatment increases oxygen vacancies^54^. In the case of non-conductive polymers, charges on different substrates using plasma treatment has been generated. Lehocký et al*.* reported that oxygen plasma treatment on polyethylene increased negative charges due to chemical reactions, creating several oxygen-based functionalities at the interface^55^. Khorasani et al*.* reported plasma oxygen treatment on plasticized polyvinyl chloride (PVC) increases negative charges and hydrophilicity^56^. Zhang et al*.* detected charge accumulation on epoxy resin using nanosecond pulsed plasma^57^. Non-conductive hydrophobic surfaces like our untreated hydrophobic glass have a tendency to absorb negative charges^58^.

In this study, we address the question: How does plasma influence the charge of sliding water drops? Is it possible to control drop charging by prior addition of a charge in the substrate by using plasma? We found that plasma treatment profoundly influences drop charging. Also, the position of the sample in either the bulk plasma or sheath area strongly influences drop charging. Sheath media phenomena are applied here to increase drop charging. Many studies on slide electrification have been carried out on glass silanated with fluorinated compounds because of the strong charge separation. For this reason, we chose glass coated with a layer of PFOTS as one sample. We varied the substrate and chose PFOTS-coated SiO_2_ and SU8 (Fig. 1b). As an example of a polymer surface, we studied polystyrene films. Exposure to low and high plasma power reveals the different effects on sliding drop charge; increasing input power intensifies plasma ionization under constant pressure and feed gas ^59–63^. Two electropositive (Ar, N_2_) gases and one electronegative (O_2_) gas were used.

The influence of plasma treatment on drop charging is a multifaceted phenomenon. It depends on the plasma configurations, the electric fields and plasma fluxes, the surface and substrate chemistries, electrical properties and so on. For this reason, our study cannot comprehensively cover the whole field and we only answer some of the arising questions.

## Materials and method

### Sample preparation

#### Substrate activation

Microscope slide glass (sodium silicate glass, Menzel GmbH, 76 × 26 × 1 mm^3^) and fused quartz (75 × 25 × 1 mm^3^, Quartz GmbH, Germany) samples were washed ultrasonically in acetone and ethanol for 10 min at 30 °C and blow-dried using nitrogen. To remove further contaminants and activate surfaces, all samples were treated by plasma (TePla, 200-G plasma system, Germany), ultrapure (99.9999%) O_2_ as a feed gas, 2.4 GHz frequency, and 30 W applied power for 35 s at 0.3 mbar. Before the gas was injected, the chamber was evacuated to 0.01 mbar, the so-called base pressure. The parameters were optimized to keep a minimum plasma charging effect on the sliding drop charge (we will discuss this in the  last section). As an alternative to plasma treatment, glass samples were cleaned in a series of experiments using the RCA (Radio Corporation of America) cleaning method.

#### RCA cleaning

First, the microscopic glass was immersed in dichloromethane for 15 min to remove organic residues from the surface. After drying using a nitrogen stream, the glass was placed into a mixture of 100 ml Milli-Q Water, 8 ml hydrogen peroxide (H_2_O_2_), and 8 ml ammonia solution (NH_3,_ 25%) and heated to 85 °C for 20 min. Afterward, it was washed three times with Milli-Q water, once with absolute ethanol, and dried in a nitrogen stream.

#### PFOTS vapor deposition

As soon as the substrates were cleaned and activated, they were placed into a desiccator (25 cm diameter, V = 9.2 L) at a 5 cm distance from the center, where 1 mL of 1H,1H,2H,2H-perfluoroctyltrichlorosilane, (PFOTS, 97%, CAS:78560-45-9, Sigma-Aldrich, Germany), was deposited on a glass Petri dish (5 cm diameter). PFOTS was selected for chemical vapor deposition because of its low boiling point of 180°C^64^. The deposition below nitrogen ambient was performed under 40 mbar for 45 min at 15–55% RH and 22–25 °C. Post cleaning with ethanol (rinsing 2 min in 500 ml) and post evacuation for 30 min at 0 mbar were performed to remove non-crosslinked PFOTS. The thickness of PFOTS layers was 3.6 ± 0.4 nm, as measured by ellipsometry (Accurion Ep4 Spektrometer, Germany).

#### SU-8 coating

Glass microscope slides (Menzel GmbH, 76 × 26 × 1 mm^3^) were sonicated in acetone (2 × 15 min) and 2-propanol (2 × 15 min) ^65^. A filtered nitrogen gun was used to dry and remove the dust particles from the glass. 1 ml SU8 (GM1060, Gersteltec Sàrl, Switzerland) was dropped onto the clean glass and spin coated. To avoid the formation of air microbubbles, the photoresist was kept in a vacuum for 10 min. The rotation speed was increased to 500 rpm, then to 3000 rpm after 5 s, and kept at this speed for 25 s. To evaporate solvents, the spin-coated layer was annealed on a hotplate at 65 °C (30 min), then at 95 °C (5 min), and again at 65 °C (30 min). Then, the sample was cooled on the hot plate until it reached room temperature. Afterwards, the samples were exposed to UV light for 8 s (290 J/cm^2^, 350–400 nm wavelength). Post-exposure baking was performed for 1 min at 65 °C and 2 min at 95 °C to thermally activate the cationic polymerization^66^. Then, unpolymerized SU8 was removed by immersing the sample in 1-methoxy-2-propanol (CAS# 108-65-6). Finally, the samples were washed for 1 min with 2-propanol to remove developer residuals and were hard baked (150 °C, 1h). The thickness of the SU8 layer was measured by Nano focus (11 µm ± 1).

#### Polystyrene coating

Polystyrene (PS) coatings on quartz substrates were prepared by dip-coating. Before coating, we cleaned the quartz substrates using an ultrasonic bath of toluene, ethanol, and Milli-Q water for 10 min. After drying by N_2_ blowing, the substrates were immersed into a solution of 1 wt% PS (molecular weight, 192 kg/mol, Sigma-Aldrich) in toluene (99.8%, Sigma-Aldrich) and then withdrawn vertically at a constant speed of 90 mm/min by a home-built dip-coater. Finally, we annealed the PS samples at 120 $\mathrm{^\circ{\rm C} }$ in a vacuum for 24 h. The thickness of the PS film on the quartz substrate was around 40 nm, measured by a profiler (P-7 stylus profiler, KLA-Tencor). The notation of surfaces is categorized based on the substrate, coating, and plasma exposure (Table 1).

**Table 1:** Plasma and coatings were used on the samples. MW stands for Microwave (2.54 GHz) and rf for radio frequency (40 kHz).

Substrate	Coating	Plasma gas (power, time, chamber frequency)	Notation
Glass	PFOTS	No plasma	Fl-glass
Glass	PFOTS	Ar (10–20 W, 15 s, MW)	ArP_MW_-Fl-glass
Glass	PFOTS	O_2_ (8 W, 6 s, RF)	O_2_P_RF_-Fl-glass
Glass	PFOTS	Ar (8 W, 18 s, RF)	ArP_RF_-Fl-glass
Glass	PFOTS	N_2_ (8 W, 18 s, RF)	N_2_P_RF_-Fl-glass
Glass + SU8	PFOTS	Ar (10–30 W, 15 s, MW)	ArP_MW_-Fl-SU8
Quartz	PS	Ar (15 W, 15 s, MW)	ArP_MW_-PS-quartz

### Methods

#### Plasma chambers for surface treatment

Two plasma chambers were utilized with different chamber volumes for two different purposes. A 10.8 L Microwave (MW) 2.54 GHz plasma source (TePla, 200-G plasma system, Germany), for plasma-bulk treatment and a 4.6 L radio frequency (RF) 40 kHz (Femto-Diener GmbH, Germany) plasma source for electrode-facing and bulk treatment. Ultrapure Ar (99.9999%, 0.3 mbar) was injected into an evacuated plasma chamber. Samples were exposed to plasma discharge with a 10–30 W output power range for 15 s. We chose Ar plasma so that the surface would not become too hydrophilic (unlike O_2_) during this short exposure time, given that slide electrification experiments require that the water drops slide off the surface. Varying the time of plasma exposure and power allowed us to systematically change the hydrophilicity of the sample. We were not able to apply electrode-facing treatment using the MW chamber because the electrode was not inside the chamber (so-called electrodeless plasma).

To take advantage of the sheath, a 40 kHz rf glow discharge plasma source with a cylindrical chamber with a 7 cm inner radius and a length of 30 cm was used. This chamber contained two electrodes, one at the top and one at the bottom. For the electrode-facing treatment, we were able to use a wide range of plasma gases (ultrapure N_2_, O_2_, and Ar 99.9999%, 0.22 mbar) and thus were able to generalize our findings for different gasses. The exposure time and output power for Ar and N_2_ were 18 s and 8 W, and for O_2_ 6 s and 8 W, so that drops were still able to slide down tilted surfaces. To take advantage of positive space charges, samples were mounted on a power electrode with ≈ 2 mm gap. However, the rf plasma source could not be used for bulk plasma treatment because the surfaces became inevitably hydrophilic.

#### Drop charge measurements

**Figure 2:**
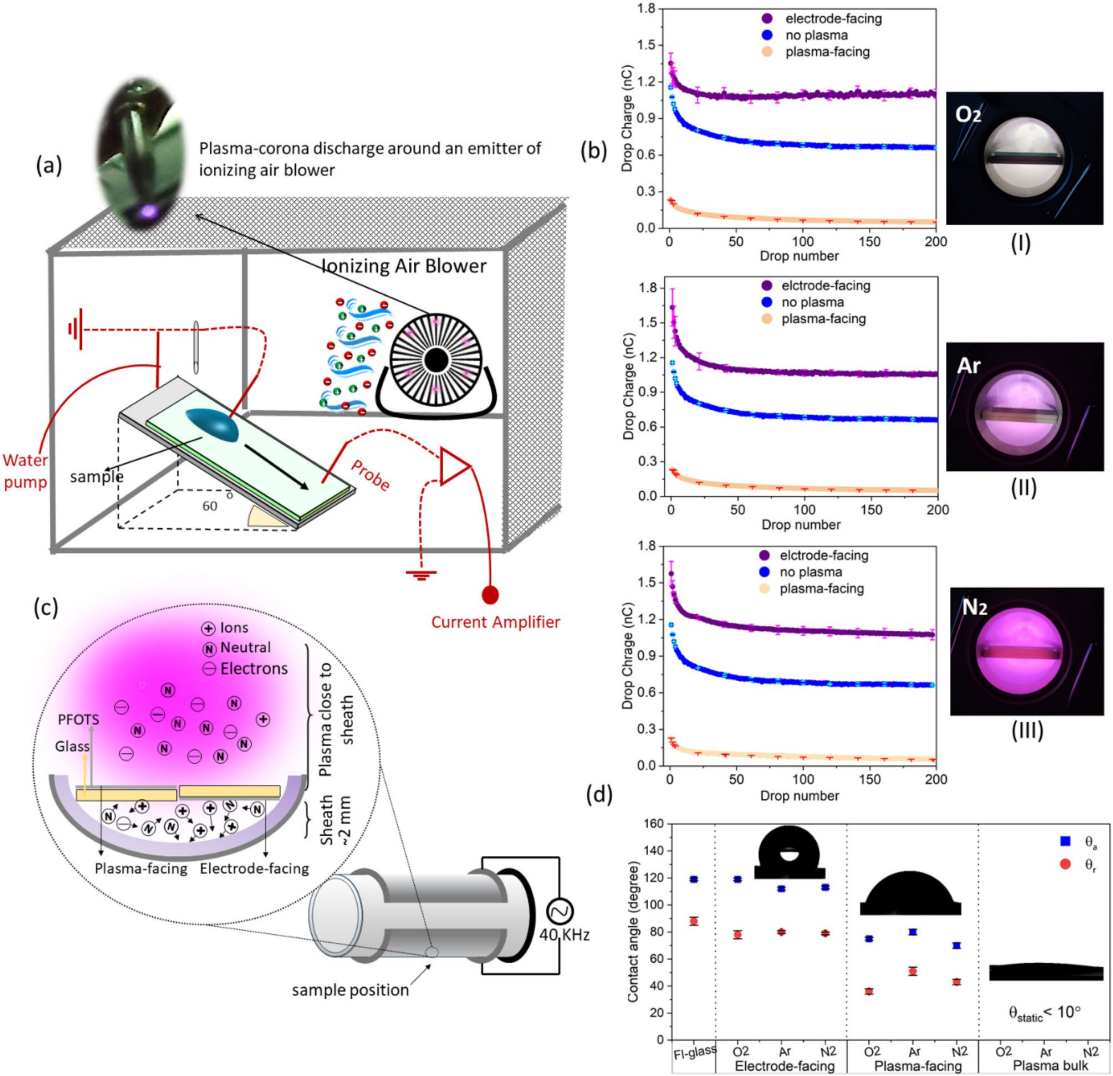
(**a**) Schematic of sliding drop electrification setup. Before the drop slide, surfaces were exposed to plasma corona discharge for neutralization (ionizing air blower with 10 emitters). (**b**) Drop charge versus drop number for Fl-glass samples before and after treatment in O_2_, N_2_, and Ar in rf plasma chamber. On the right, optical images of plasma glow discharges for O_2_, Ar, and N_2_ are shown. The conditions were: I) O_2_ (6 s, 8 W, 0.22 mbar), II) Ar (18 s, 8 W, 0.22 mbar), III) N_2_ (18 s, 8 W, 0.22 mbar). Dark blue, cream, and purple color data points represent the drop charge of pristine Fl-glass, Fl-glass faced to plasma, and Fl-glass faced to the electrode respectively. Light colors are error bars per 20 drops. (**c**) Schematic indicating the location of Fl-glass in the rf plasma chamber. The PFOTS-coated side of the glass either faced the electrode or the bulk plasma. (**d**) Advancing and receding contact angles before and after treatment. Transition from a hydrophobic state using electrode-facing to hydrophilic and super hydrophilic using plasma-facing and plasma bulk.

To measure the charge of a series of water drops, the plasma treatment samples were mounted on a grounded metallic plate at a tilt angle of 60° (Fig. 2a). Residual electrostatic charges were neutralized for 10 min by a corona discharge of an ionizer. 45 µL of ultrapure water drops, 18.2 MΩ.cm resistivity (Sartorius, arium®pro, Germany) were ejected from a grounded syringe (stainless steel Hamilton syringe needle, 2 mm diameter) onto the top of the tilted plate. Due to variations in temperature between different days of the year, fatigue of the tube in the peristaltic pump, resulting changes in density and surface tension and the error in calibration of the balance we estimate the absolute error to be ± 1 µL. Within one series of drops the statistical variation in drop volume was only ≈ 0.1%. The needle was 5–8 mm above the surface to allow the drop to detach from the needle. The syringe was connected to a peristaltic pump (Gilson Minipuls 3, Wisconsin, USA). The time between drops was 9 ± 1 s, if not otherwise mentioned. After landing on the sample, the drops began to slide. They immediately contacted a grounded electrode and then moved on. When the drops had moved 4 cm after the grounded electrode, their charge was measured by the second electrode, which was connected to a low noise current amplifier (response time: 0.8 ms, DDPCA-300, FEMTO, Germany). The drop charge was obtained by integrating the first 0.1–2.5 ms of the current^3^. An benchtop ionizing air blower, which is a plasma corona discharge^53^ device (Simco-Ion, Aerostat PC, USA), was placed a constant 20 cm from the samples and allowed to neutralize the surface for 10 min before measuring the sliding drop charge. The estimated flow rate was 1.4 m^3^/min. The ionizer produces positive and negative ions (alternating current AC) by discharging 10 sharp needles (emitter^67^).

#### Contact angle measurement

Contact angles were measured with sessile drops and side view imaging (Dataphysics, SCA20, Germany, Zoom factor 1.0). The operating drop volume was increased and decreased at a constant rate of 0.5 μL/s between 6 and 70 µL. Advancing (θ_a_) and receding (θ_r_) contact angles were obtained by fitting the contour of drops obtained from side-view videos by an ellipse fitting during inflation and deflation, respectively. Care was taken to localize the needle on the back of the droplet to reduce distortion of the drop surface caused by the needle. In some cases that ellipse fitting could not follow the drop curvature, polynomial fitting was utilized due to its flexibility^68^.

#### Zeta potential measurement

The zeta potential ζ of pristine and plasma-treated surfaces was obtained (SurPASS 3, Anton Parr, Austria) over a macroscopic sample size (20 × 10 mm^2^). Conductivity of the electrolyte solution was adjusted by KCl (0.001 mol/l) in ultrapure water (resistivity 18.2 MΩ.cm), and 0.05 mol/l HCl and 0.05 mol/l KOH was used for acid and base pH titration. Before every measurement the pH probe was calibrated by 3 different buffer solutions (pH 3, 7, 10), and conductivity probe was calibrated in 0.1 mol/l KCL.

#### Surface work function measurement

The work function ($\varphi$) of PFOTS and plasma-treated samples on an N-type Si (111) substrate was determined based on Kelvin potential measurements, measured by a single-point Kelvin probe (Anfatec, AFT-KPTT, Germany) in ambient air (operating humidity 40%, temperature: 24 °C). The base plate and the Kelvin probe-head surfaces were coated with gold. The base plate, sample holder, and sample were grounded. Before starting, the probe calibration was assessed using freshly cleaved highly oriented pyrolytic graphite (HOPG), a reference with a known work function of *φ*_ref_ = 4.46 ± 0.03 eV. The work function was measured at five different points on every sample. The work function of the test sample was obtained by $\varphi ={\varphi }_{tip}-eUK$, where ${\varphi }_{tip}={\varphi }_{ref}+e{UK}_{ref}$. Where UK is the Kelvin potential. The detected current in the setup increased with the sensor size (1.4 mm), oscillation amplitude, and applied bias voltage. To measure the Kelvin potential independent of the distance, the tip was placed close to the sample in order to reach a certain distance (less than 1 mm). The Z-axis distance was monitored and kept constant by a software-controllable stepper motor. The work function measurement of a sample was taken both before and after Ar plasma treatment. The error bars were obtained by averaging three samples. For example, the data corresponding to point number four is derived by averaging measurements from three samples, with the fourth point being included in the calculation.

#### X-ray photoelectron spectroscopy

Photoelectron XPS spectra (Kratos, Axis Ultra DLD, UK) were acquired in a UHV chamber (base pressure 10^−10^ Torr) using a monochromatic Al-Kα X-ray source operating at hν = 1486.6 eV, 15 kV voltage bias and 10 mA emission current. Based on calibration with Agd5/2, the source line width was ≈ 1.0 eV FWHM. To determine the quantitative elemental composition, survey spectra were acquired in the 0–1200 eV binding energy range at pass energy of 80 eV (step: 1 eV). Shirley's function was the criterion for subtracting the background. The high-resolution spectra aimed to accurately determine the binding energy levels and states of the topmost elements. High-resolution at pass energy of 20 eV (step: 0.1 eV) spectra were obtained for the F 1s, O1s, C 1s, and Si 2p core-levels. The duration of each scan was limited to about 1.5 min (at each measurement point, 10 scans were recorded and averaged). The spectral acquisition of three regions has been indicated above. Peak positions were referenced to the silicon single crystal (100) Si 2p line at 99.3 eV. Gaussian/Lorentzian (GL) distributions were used to fit the peaks. Curve fitting and data analysis were done by CasaXPS V23.16.

#### Dielectric spectroscopy

A Novocontrol Alpha frequency analyzer, consisting of a broadband dielectric converter and an active sample head, was used. Samples were measured with two stainless steel electrodes with a diameter of 20 mm. A broad frequency range from 10^−2^ to 10^7^ Hz was employed. All measurements were conducted at ambient temperature. The applied voltage had an amplitude of 1 V.

## Results and discussion

### Drop charge versus drop number on untreated surfaces

Before plasma treatment, the first drop always carried the highest electrical charge (Fig. 2b blue symbols). Subsequently, the charge of the drops decreased with the number of drops, reaching saturation after 20–65 drops. The drop charge increased as the drop time interval increased ^5,69^. Hydrophobic surfaces become negatively charged when they come into contact with water, possibly because they adsorb hydroxyl groups. The hypothesis is that the first drop of water leaves negative charges behind when it slides over the surface^5^. Consequently, the drops acquire positive charge. The deposited charges are attributed to leftovers from the electric double layer. The second and subsequent drops deposit fewer negative charges, resulting in lower positive charges compared to the first droplet. Over a timescale of minutes, surface charges are neutralized. It is not yet clear how surface charges are neutralized. It could be by electron or ion transport in the sample, or by ions in the air, depending on the specific conditions.

### Tuning drop charge by plasma-and electrode-facing treatment

To tune the plasma-assisted surface chemistry and charge, samples were mounted on the power electrode of the rf (40 kHz) plasma chamber to take advantage of the positive space charge of the sheath^70^. Two electropositive (Ar, N_2_) gases and one electronegative (O_2_) gas were used (Fig. 2b I–III). Two Fl-glass samples were positioned at identical positions but with different orientations (Fig. 2c). One had its PFOTS coating side facing the power electrode (referred to as the electrode-facing sample). The other had its coated side facing the bulk plasma (plasma-facing sample). While keeping the operating parameters for the three gases constant (0.22 mbar pressure, 8 W power), we adjusted the exposure times (6 s for O_2_, 18 s for N_2_ and Ar). In the case of O_2_ plasma, the exposure period could not be increased above 6 s, because after longer periods the surface became hydrophilic, and drops would no longer slide.

Drop charges increased when the samples were electrode-facing (Fig. 2b purple points). Drop charges decreased when the sample were plasma-facing (Fig. 2b cream points). The first water drop running down an electrode-facing-treated sample carried a charge of 1.6 ± 0.1 nC after Ar and N_2_ treatment (ArP_RF_-Fl-glass and N_2_P_RF_-Fl-glass). After oxygen plasma treatment, the first drop had a lower charge of 1.3 ± 0.1 nC (O_2_P_RF_-Fl-glass). The saturation value of all three samples increased from 0.69 ± 0.01 nC (no plasma) to 1.1 ± 0.03 (Fig. 2b). Our criterion to classify drop charge as “saturated” is that the average difference between subsequent drop charges was less than 0.01 nC. Based on this criterion the charge of the 30th drop of O_2_P_RF_-Fl-glass, 43rd drop of ArP_RF_-Fl-glass, and 60th drop of N_2_P_RF_-Fl-glass, and all subsequent drops, had reached saturation. In contrast, plasma-facing treatment reduced the saturation to a relatively low value (0.07 ± 0.01 nC). The error bars are the variation between three measurements with different samples. When the first sample was being measured the rest were kept in a vacuum. The increase (1st drop ~ 71%, saturation ~ 64%) in drop charge after electrode-facing treatment of Fl-glass and the decrease (first drop ~ 82%, saturation ~ 99%) after plasma-facing treatment of Fl-glass were observed for all three gases. We attributed the increasing drop charges after electrode-facing treatment to the positive space charge in the sheath region. The effect was very different for the two surfaces. On the electrode-facing treatment the advancing and receding contact angles only decreased by 3°–10°, while for the plasma-facing treatment the advancing and receding contact angle decreased by around 45°, 60° respectively, compared to pristine Fl-glass (Fig. 2d). In general, Fl-glass undergoes a transition from a hydrophobic to a super hydrophilic state when it is moved away from the electrode (sheath which is non-neutral region) and towards the plasma bulk (quasi-neutral). Probably the plasma-facing sample was situated in a transition region between the sheath and bulk plasma ^70^, which prevented it from becoming too hydrophilic, as is the case in the bulk of the rf chamber.

### Surface characterization of plasma and electrode-facing treated samples

To find out how plasma effects drop charging we characterized samples before and after O_2_ plasma treatment.

#### Zeta potential measurements

**Figure 3:**
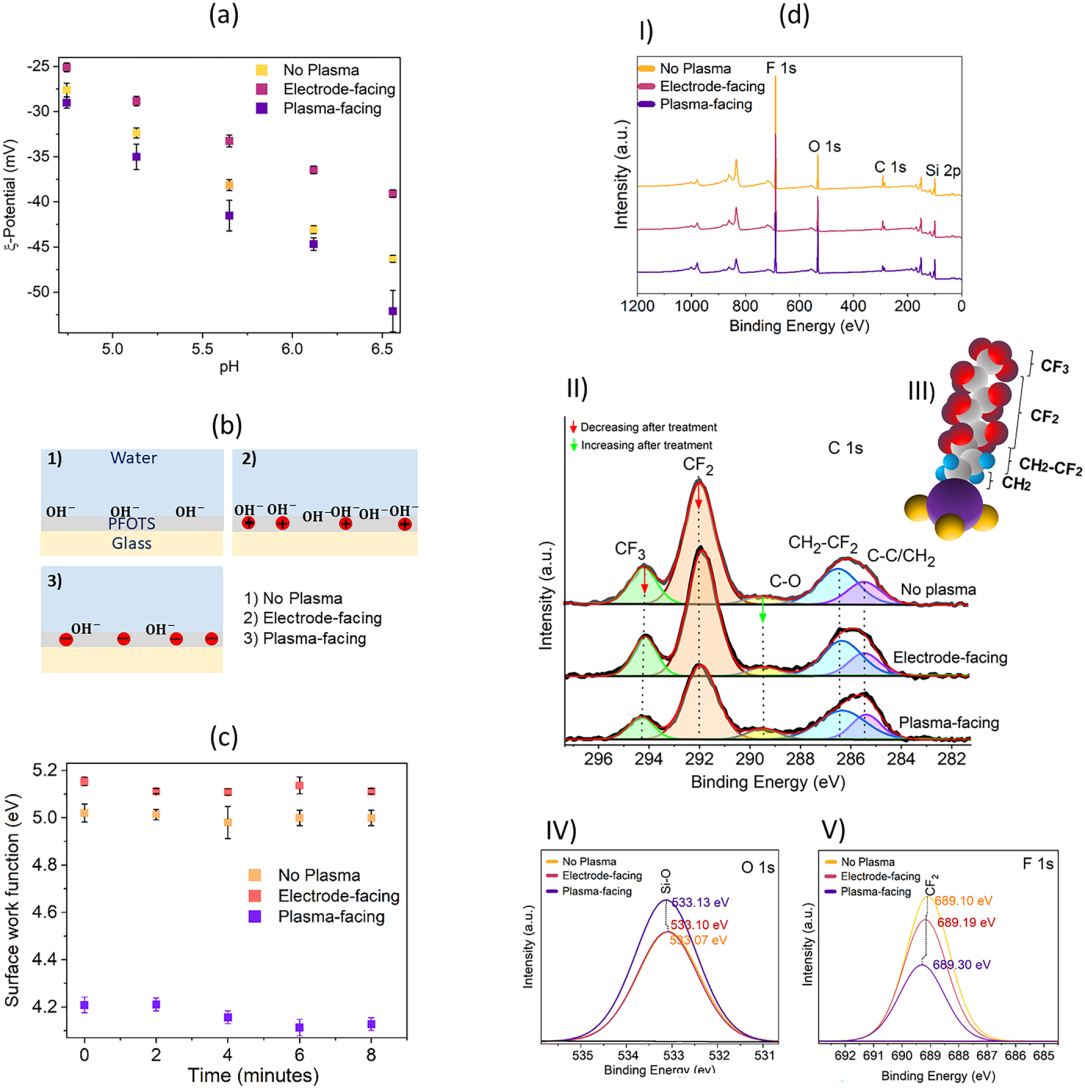
Surface characterization of PFOTS on glass and Si-wafer after plasma-facing and electrode-facing treatment (6 s, 8 W, 0.22mbar, feed gas: O_2_). (**a**) Zeta-potential versus pH for untreated, electrode-facing, and plasma-facing -treatment of Fl-glass surfaces. (**b**) Schematic of how the plasma-deposited charge influences the OH- concentration at the water-PFOTS interface. (**c**) Surface work function measured by Kelvin probe on Fl-Si surfaces. (**d-I**) XPS survey spectra, (**d-II**) Evolution of the C 1s region of pristine Fl-Si after electrode and plasma-facing. (**d-III**) Schematic of CF_3_(CF_2_)_5_(CH_2_)_2_-Si (PFOTS) exhibiting the different contributions for the C 1s line. (**d-IV, V**) Evolution of the O 1s and F 1s regions for pristine PFOTS after electrode and plasma-facing.

In the pH range of 4.0–6.5, the zeta potential showed that the electrode-facing surface became less negatively charged compared to untreated Fl-glass (Fig. 3a). In contrast, the plasma-facing side became more negative.

We interpreted these results in the following way. Usually, the negative zeta potential is attributed to the enrichment of hydroxyl ions. At the rear of the contact line some of these hydroxyl ions remain on the surface. As a result, the Fl-glass becomes negatively charged while the cationic H_3_O^+^ remain in the drop, resulting in a positive charge. We can explain the increased drop charge when the surface is exposed to the sheath (electrode-facing treatment) as follows: the positive ions in the sheath accelerate towards the polymer. As a result, the surface acquired positive ions or surface groups, which partly compensate the negative surface charges. Therefore, the saturation drop charge of these samples was much higher, i.e., it reached 1.2 nC. Given this, we propose that positive ions were deposited on the top layer of the surface (Fig. 3b). These positive ions allowed more hydroxyl ions to adsorb at the water-PFOTS layer, because they reduced the repelling electric potential. Consequently, when a drop slides over the surface, more hydroxyl ions can remain on the top layer of the surface and the positive drop charge increases. The surface chemistry does not seem to change substantially, otherwise, the contact angles would have changed more.

In contrast, the plasma-facing, and the plasma bulk exposed surfaces both led to a substantial change in the surface chemistry and the contact angles and drop charge decreased. Lower contact angles led to less charge transfer at the rear contact line ^71^. In addition, negative ions may be deposited in the top layer of the surface and cause the opposite effect, meaning they prevent hydroxyl ions enriching at the interface.

We are not yet able to determine where and how the ions are deposited in the top layer. We are able to distinguish them from the surface charges deposited by the drop because they are more persistent. While the surface charges are neutralized within seconds or minutes, the ions in the top layer remain even after days.

#### Kelvin probe measurements

To support our perception of how plasma treatment alters the surface charge, we conducted Kelvin probe measurements (Fig. 3c). We used naturally oxidized Si wafers as substrates. Due to their conductivity, Kelvin probe measurements on Si wafers are better defined than on thick insulating samples. The surface work function of pristine Fl–Si was + 5.00 ± 0.03 eV. Plasma-facing treatment led to a less positive work function + 4.2 ± 0.02 eV. It was most likely caused by adding surface groups which tend to acquire negative charges.

In contrast, electrode-facing treatment increased the work function to 5.12 ± 0.02 eV. The work functions of pristine and electrode-facing samples were stable for at least a few hours after treatment. These measurements confirm that positive ions are deposited by the plasma when the sample faces the electrode side.

#### XPS measurements

To analyze changes in surface chemistry, XPS measurements were carried out. Four intensive photoemission zones were detected in survey spectra (Table S1, Fig. 3d-I). The O/F ratios for pristine, electrode and plasma-facing were 0.4, 0.48, 1.07. These four zones in high resolution spectra were at 685–693 eV (associated with F 1s photoelectrons), 530–536 eV (O 1s), 282–297 eV (C 1s), and 97–106 eV (Si 2p). Plasma-facing treatment increases the oxygen content and decreases the amount of fluorocarbons CF_2_, CF_3_, C–C/CH_2_ (Fig. 3d-II-IV, Table 2) in PFOTS. The sketch of the PFOTS molecule is illustrated in Fig. 3d-III. The details of probable mechanisms of plasma-PFOTS interaction are included in the Supporting Information.

**Table 2:** The peak area of the bonding components obtained from C 1s, F 1s, O 1s, Si 2p core-level spectra.

Component	Binding energy (eV)	Sample treatment		
		No plasma	Electrode-facing	Plasma-facing
C 1s				
C–C/CH_2_^73,84^	285.2	1965	1842	1911
CH_2_-CF_2_^73^	286.2	3679	3625	3567
CO^50,72^	289.2–289.8	712	734	886
CF_2_	291.8	9942	9798	6370
CF_3_	294.1–294.3	2586	2430	1467
F 1s				
CF_2_	689.1–689.3	188,397	158,807	102,808
Si 2p				
Si	99.3	9988	10,338	11,838
SiOH	99.9	5193	5375	6156
SiO_2_	103.6–103.7	6891	7030	7103
O 1s				
Si–O	533.1	54,262	52,630	68,892

A short and wide peak in 289.2 eV can be attributed to a C-O fragment (CO_2_H^50^/RCOO^72^). In pristine PFOTS, the C–O to C–C/CH_2_ ratio was 0.3. After electrode and plasma-facing treatment, the ratios increased to 0.4 and 0.46, respectively. The decrease in the C–C/CH_2_ peak area and increase in C–Ocould indicate that electrode and plasma-facing treatment dissociate carbon bonds. Some of these broken bonds oxidized, while the remaining bonds were gasified. The amount of C–O formed was smaller than the amount of C–C/CH_2_ dissociated.

Furthermore, the pressure in the rf plasma chamber increased by 0.04 mbar during the treatment. We assume that part of the organic layer was converted into gas. The Si–O bonds (533.1 eV, Fig. 3d-IV) in the electrode-facing treatment at the peak area of Si–O decreased by about 3% compared to that of the pristine sample. The Si–O peak increased by about 27% on the plasma-facing side, indicating substantial etching. In contrast, the intensity on the electrode-facing side was negligible. The results support those already obtained for the Si peak. In the Si 2p region, a sharp peak corresponds to uncharged elemental Si (99.3 eV), Si–OH (99.9 eV), and a Si^4+^ in SiO_2_ (103.66–103.72 eV) (Fig. S1). The hydroxyl groups bonded to Si in the 99.9 eV increased 5% and 20% on electrode and plasma-facing, respectively, making the surface more hydrophilic and polar. The ratio of the total peak area of F 1s (Fig. 3d-V) to Si 2p was an indication of the coverage of the self-assembled layers^73^. The F 1s/Si 2p peak ratio on the pristine sample was 8.6. After electrode-facing treatment, it decreased slightly to 7.2. A substantial decrease to 4.1 was observed after plasma-facing treatment. This demonstrates that the presence in the plasma region leads to a reduction of PFOTS. However, there was no significant decrease in PFOTS coverage in the electrode-facing treatment. Plasma-facing treatment may lead to more de-coverage, potentially increasing the chance of Si radicals chemically reacting with oxygen. A similar reduction of PFOTS was observed using a solvated electron soluton^50^.

In summary, the peak area of fluorinated components (CF_3_ and CF_2_) differed slightly in electrode-facing versus pristine samples, but they decreased considerably in plasma-facing experiments. As a result of oxygen introduction, the summation of the peak area of the polar components (Si–O, Si–OH, C–O) showed that pristine PFOTS has a higher polarity than that of electrode-facing. This finding is in line with zeta potential and Kelvin probe results.

### Effect of plasma bulk-surface treatment on drop charge

**Figure 4:**
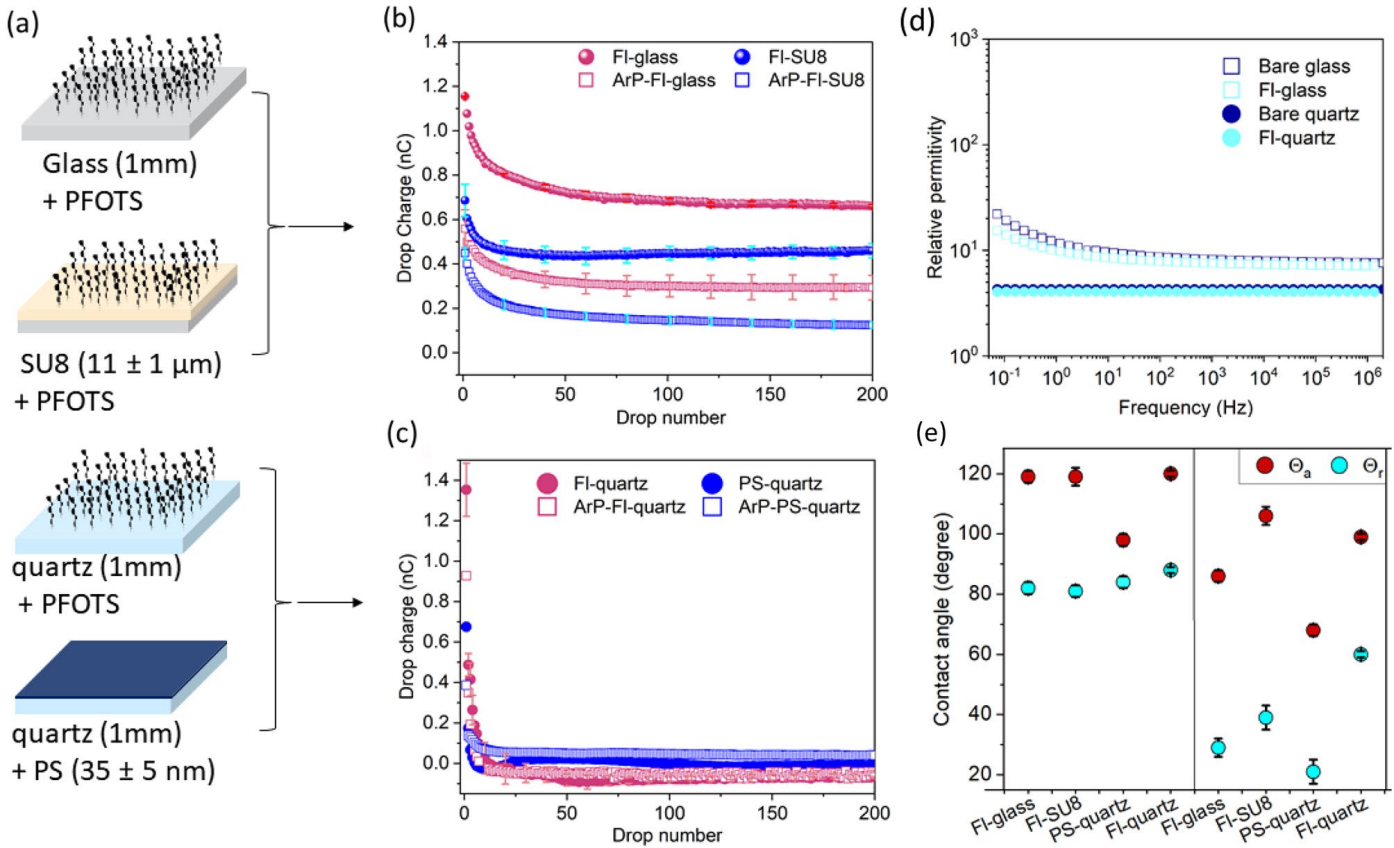
Effect of substrate and Ar bulk plasma-surface treatment on slide electrification. (**a**) Schematic of samples that were used. (**b**) Drop charge versus drop number on Fl-glass, Fl-SU8 before (filled circular red and blue) and after plasma treatment (15 W, 15 s) (hollow square red and blue). The maximum error bar was 0.1 nC (bright colors). (**c**) Drop charge versus drop number for PS-quartz and Fl-quartz before (filled circular red and blue) and after plasma treatment (hollow square red and blue). (**d**) Dielectric spectroscopy measurement of glass and quartz before and after PFOTS deposition. (**e**) Advancing (θ_a_) and receding (θ_r_) contact angles of the four samples before and after plasma treatment.

In further studies on the effect of bulk plasma, we carried out experiments with the MW plasma chamber. We only used Ar gas, because drops did not slide on samples with N_2_ and O_2_ plasma exposure. The drop charge was measured on Fl-glass, Fl-SU8, Fl-quartz, and PS-quartz. We altered the substrate and the coating material once on each sample (Fig. 4a). The first two samples were deposited with the same chemical (PFOTS) and different substrates (glass and SU8) (Fig. 4b). The second two samples had identical substrates (quartz) but different coatings (PFOTS, PS) (Fig. 4c). Plasma-treated samples were denoted by adding the shortcut ArP, e.g., ArP-Fl-glass.

When applying a 15 W Ar bulk plasma for 15 s, the first drop charge on ArP-Fl-glass and ArP-FI-SU8-glass was only 25–40% of the first drop charge on the pristine sample (Fig. 4b). The saturation charge decreased to roughly 55% of the saturation charge of the pristine samples.

These results agree with the observations in the plasma-facing sample in the rf chamber.

#### Substrates effect

For the first drop, the charge was independent of the substrate. For all subsequent drops, however, drop charges on Fl-quartz were much lower (Fig. 4c) than on Fl-glass and Fl-SU8. In addition, the drop charge versus drop number (Q-vs-n) saturated after 7–14 drops, much faster than on glass or SU8 (Fig. 4b). Except for the first few drops, drop charges were in the order of < 0.02 nC. For the pristine sample, the steady state drop charge was slightly negative (≈ − 0.02 to − 0.05 nC).

The effect of plasma on quartz samples (Fig. 4c) was minor (< 0.02 nC), and it led to a decrease of the absolute value of the drop charge. PS-quartz and Ar-PS-quartz changed in the same order of magnitude (< 0.1 nC). The low saturation drop charges on quartz demonstrate that slide electrification is sensitive to the substrate below the hydrophobic top layer.

We assume that the long neutralization time is the reason for the fast saturation and low drop charge on quartz. Fused quartz with a resistivity of the order of 10^17^ Ωm is less conductive than sodium silicate glass (electric resistivity 10^8^–10^12^ Ωm). Thus, surface charges are only neutralized slowly, and saturation is reached after ≈ 10 drops. To support this hypothesis, we measured the dielectric spectra of the materials. Glass has a higher dielectric constant than quartz (Fig. 4d). Furthermore, the relative permittivity depends on the applied frequency; slow relaxation processes lead to an increasing permittivity at a frequency below ≈ 10 Hz.

To explain why Q-vs-n curves on glass and SU8 change qualitatively after bulk plasma treatment, we deduced that negative ions are deposited in the top layer of surfaces. We assumed that this charge addition is more effective on glass and SU8 than on quartz for two reasons: First, the atomic lattice is less stable than in quartz, and it is easier to build in a defect. Secondly, the electrostatic self-energy of an ion in glass or SU8 is lower than that on quartz due to the higher dielectric permittivity. The electrostatic self-energy of a unit charge *e* is $E={e}^{2}/\left(8\pi \epsilon \varepsilon {\varepsilon }_{0}R\right)$. Here, *ε* is the relative dielectric permittivity of the material, *ε*_0_ is vacuum permittivity, and *R* is the ionic radius. The dielectric permittivities of quartz and sodium silicate glass are 3.6 and 7.9 at 1 MHz, respectively.

Measurements of θ_a_ and θ_r_ were undertaken on the four samples to investigate alteration in the uppermost layer and its connection with the change in contact angles. The Fl-glass and Fl-SU8 showed similar contact angles of θ_a_ = 118° ± 2 and θ_r_ = 82° ± 2. The Fl-quartz advancing contact angle (θ_a_ = 118° ± 2) was similar to that of the other samples, but its receding contact angle was slightly higher (θ_r_ 88° ± 2) (Fig. 4e, left side). On polystyrene, the advancing contact angle was 24° lower than on the fluorinated surfaces. The PS-quartz surface exhibited the lowest contact angle hysteresis. After plasma treatment (15 W, 15 s), all contact angles decreased (Fig. 4e, right side). After plasma treatment, both the advancing and receding contact angles of all samples decreased drastically. The differences in each advancing and receding angle, before and after treatment, are as follows: Fl-glass: ∆θ_a_ ≈ 32° and ∆θ_r_ ≈ 52°, Fl-SU8: ∆θ_a_ ≈ 12° and ∆θ_r_ ≈ 52°, PS-quartz ∆θ_a_ ≈ 20° and ∆θ_r_ ≈ 63°, Fl-quartz: ∆θ_a_ ≈ 24° and ∆θ_r_ ≈ 21°). The θ_r_ decreased even more than advancing contact angles, so the contact angle hysteresis increased. The absolute values of the contact angles depend on the power and duration of argon plasma treatment. Thus, the surface chemistry of the samples became more polar. Plasma treatment did not impose notable surface roughness, i.e., surface roughness changed in the order of error bars (≈ 0.4 nm) (Fig. S2).

### Effect of plasma-power on drop charge of Fl-SU8

**Figure 5:**
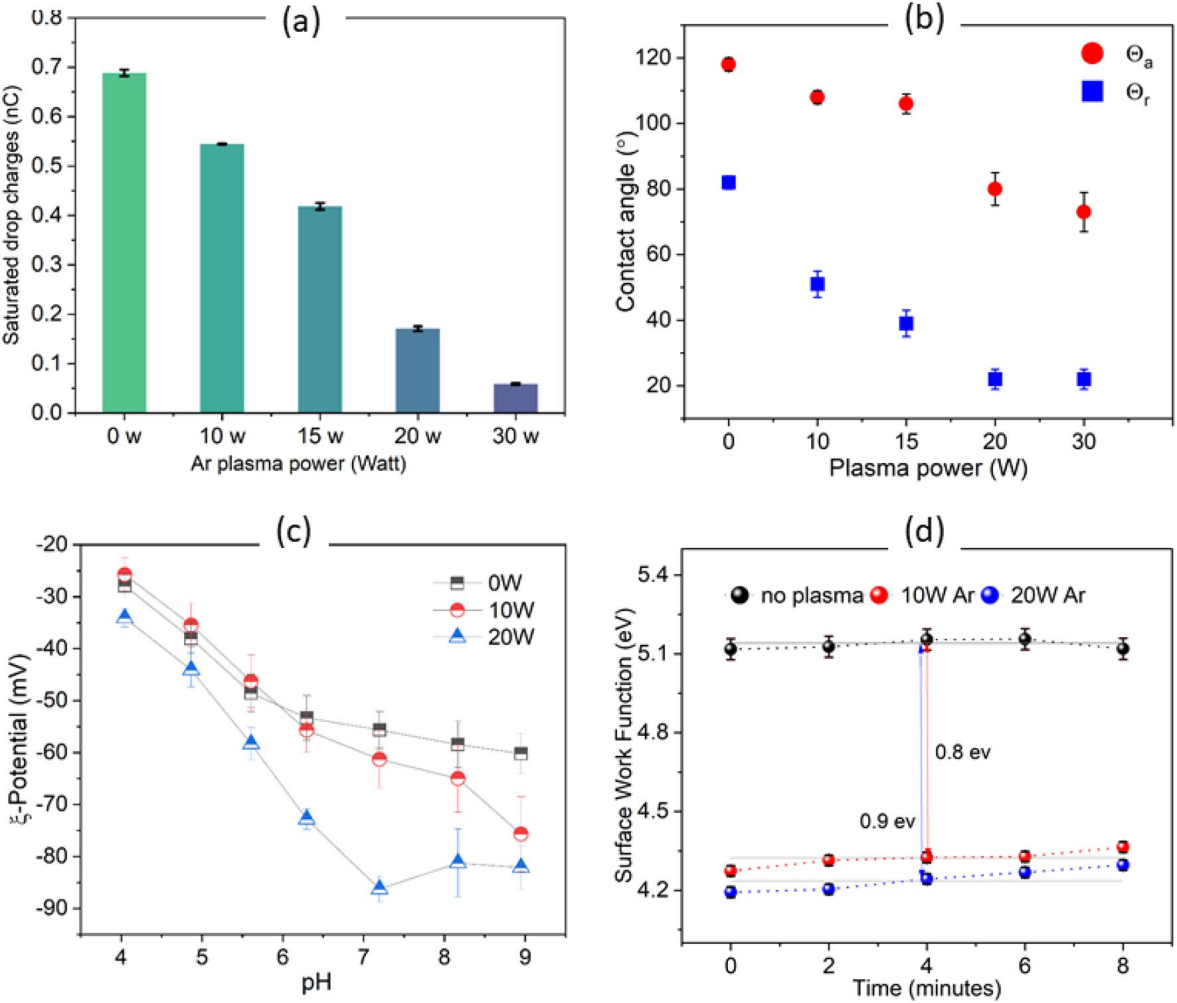
Surface characterization of Fl-SU8 and Fl-Si after Ar plasma treatment (MW plasma chamber, 10–30 W, 0.3 mbar, 15 s). (**a**) Drop charge saturation after plasma treatment of fluorinated SU8 surfaces, ArP_MW_-Fl-SU8. The bar chart shows the mean charge of the last 200 drops. (**b**) Advancing and receding contact angles as a function of plasma power. (**c**) Zeta-potential versus pH for pristine Fl-SU8 (0 W) and ArP_MW_-Fl-SU8 in 0.001 mol/l KCL solution. (**d**) The work function of plasma treated Fl-Si was evaluated at five different locations, maintaining a measuring interval of 2 min between each spot.

We chose one type of surface as an example to study how the plasma power influences drop charging. We varied the power of Ar plasma in the MW chamber for Fl-SU8 and kept the interval constant at 15 s. Fl-SU8 (with 90 min CVD) was chosen because water could slide off the surface even after 30 W plasma power treatment (unlike Fl-glass, PS-quartz, and Fl-quartz, where the drops did not slide anymore). The drop charge increased as the drop time interval increased^5^. This time increase enabled us to detect a low current signal of drops after 20 W plasma treatment. Drop charges of ArP_MW_-Fl-SU8 decreased as plasma power increased (Fig. 5a). In parallel, the contact angles decreased (Fig. 5b). This decreasing drop charge is most likely caused by the low receding contact angles. Low contact angles cause low charge transfer from the electric double layer to the free surface^71^.

Increasing plasma power led to an increasingly negative zeta potential of the surface (Fig. 5c). Particularly, for pH> 6, the zeta potentials for the plasma-treated surfaces were more negative than those of the pristine sample. This observation indicates that acidic groups are formed on the Fl-SU8 surface by the plasma treatment. In general, materials that come into contact with bulk plasma will charge negatively. Given that electrons in the plasma are more mobile than ions (higher temperature, lower mass), their flux is substantially bigger^74^.

In parallel with the decreasing zeta potential, the surface work function decreased (Fig. 5d). Here, Fl-Si instead of Fl-SU8 was used to ensure the sample grounding for the Kelvin probe measurements. A surface work function of ϕ = 5.12 ± 0.04 eV was observed on pristine Fl–Si. It decreased to 4.27 eV and 4.19 eV upon 10 and 20 W Ar plasma exposure, respectively. The decrease of ≈ 0.9 eV (average of 5 points) after plasma exposure indicates that negative charges are added on the surface by plasma^75^. The accumulation of negative electric surface charges after plasma treatment has been observed on polymeric surfaces^45,76–78^. The Kelvin tip was held in five different positions on Fl–Si for 2 min after and before plasma treatment. The work function of Fl–Si remained constant within experimental accuracy, however, the ArP_MW_-Fl–Si sample had a gradual increase. An increase in the work function means the plasma deposited charge on the fluorinated surface was dissipated. Zhang et al. found that the decay surface charge of plasma treatment of epoxy resin occurred rapidly within the first 10 min^57^. Accordingly we tracked the surface work function at the same time scale which had the fastest charge dissipation. Indeed, plasma-surface charge dissipation naturally happens by air for long-term storage, which is explained in the next section.

After 30 W plasma treatment of ArP_MW_-Fl-SU8 the drop saturation charge was reduced to 0.05 nC. We could partially recover the drop charge by annealing at 70 °C or 230 °C. After annealing the saturation drop charge increased to 0.15 nC and 0.25 nC, respectively (Fig. S3). Also, the contact angles partially recovered. We believe that this recovery of contact angles and drop charge is caused by neutralization of the plasma-induced surface charges. Indeed, the chemical aspect of plasma treated surface after heating changes^79,80^ in parallel to charge.

### Long-term influence of treatment on drop charging

**Figure 6:**
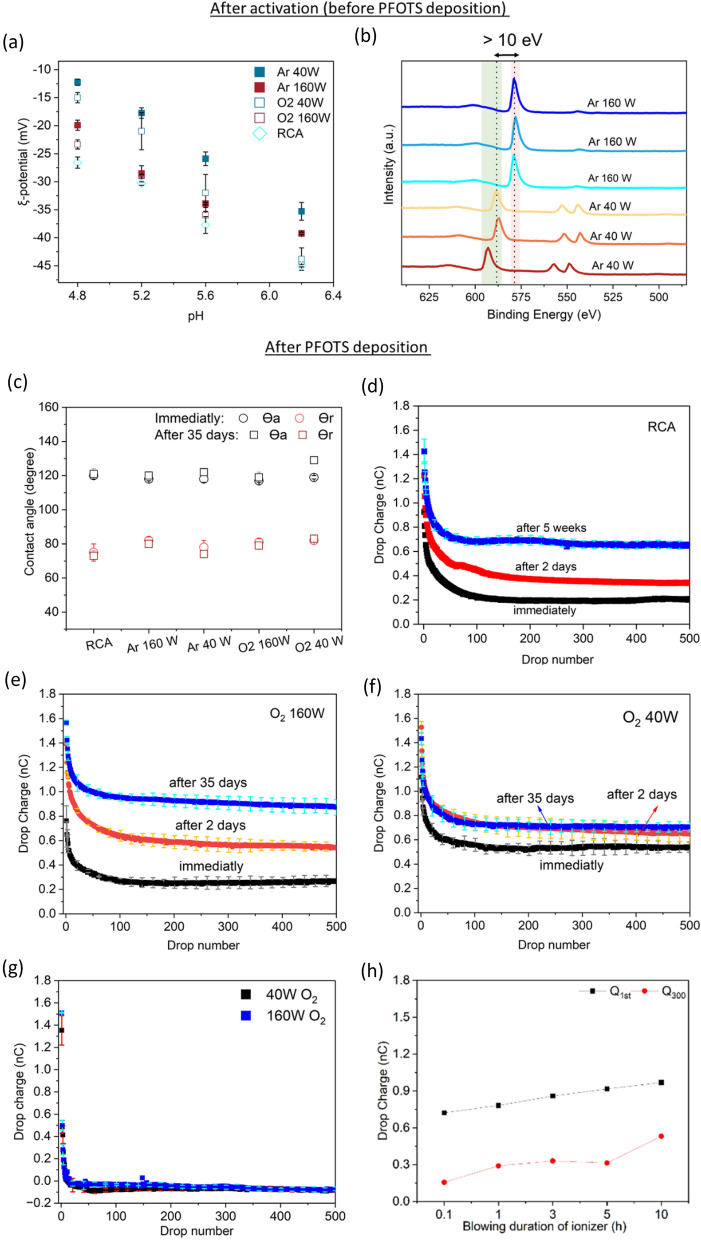
Effect of MW plasma-glass surface treatment vs power (**a**, **b**), and representative Q-vs-n curves showing the influence of plasma and RCA activation of glass and quartz with subsequent PFOTS vapor deposition on slide electrification (**c**–**h**). (**a**) Zeta-potential versus pH value for pristine glass activated by plasma (40 W and 160 W, 35 s, working gas: O_2_, Ar) and RCA. (**b**) XPS survey spectra of different glasses (the binding energy range of O1s was selected) after 40 W and 160 W Ar plasma-glass activation. All spectra were acquired without using electron flood gun neutralizer. (**c**) Advancing and receding contact angles of Fl-glass vs different glass activation, θ_a_ remained constant (118° ± 1°), and θ_r_ varied by a maximum of 5˚. After 35 days θ_a_ increased by up to 8° and θ_r_ varied by 3°. (**d**–**g**) The sliding drop charge of the 500 drops on Fl-glass and Fl-quartz activated using RCA (**d**), 40 W and 160 W O_2_ plasma (35 s, 0.3 mbar) on Fl-glass (**e**, **f**) and on Fl-quartz (**g**). Samples were measured immediately after silanization (dark black data points with light black error bars: 0.05 nC in average, the time interval between successive drops: 9 ± 1 s), after two days storage (dark red data points with light red error bars: 0.07 nC), and after five weeks storage (dark blue data points with light blue error bars: 0.05 nC in average). Samples were stored in a laboratory box in ambient air with humidity around 45–60% at 22–26 °C.

Up to here, we have reported about the influence of plasma treatment on coated surfaces. However, surfaces usually need to be activated before they are deposited with silanes. We aim to determine whether plasma surface activation before PFOTS deposition influences drop charging. MW plasma treatment reduces the zeta potential of glass (Fig. 6a). Samples with higher plasma power showed a more negative zeta potential than those with lower power. The negative sign after RCA and plasma activation agrees with what has been observed before on Si-wafer^81^.

Moreover, XPS survey spectra (in range of O1s) showed that the 160 W Ar plasma-glass treatment causes a 10 eV shift toward lower binding energies as compared to the 40 W treatment (Fig. 6b). One possibility is that the intense plasma activation added valence electrons to the glass, causing a decrease in the binding energy. Similar red shifts were observed with longer plasma exposure on insulator surfaces^45^. In principle, during X-ray-sample interaction, “positive charging on the sample during X-ray retards outgoing electrons and tends to make the peaks appear at higher binding energies, whereas excessive charge compensation can make the peaks shift to lower binding energies”^82^.

Immediately after activation, the PFOTS deposition was carried out. The contact angles of Fl-glass for all pretreatments were similar (θ_a_ = 117°–120°, θ_r_ = 75°–82°, Fig. 6c). Contact angles also remained constant over time; five weeks after deposition no significant change was detected. Additionally, the surface roughness of Fl-glass remained constant within experimental accuracy immediately after sample preparation, e.g., for 40 W and 160 W Ar plasma activation (in 1 × 1 µm^2^: RMS = 1.4 ± 0.3 nm, 1.2 ± 0.2 nm, respectively) (Fig. S4). The relatively constant contact angles of Fl-glass surfaces indicated that the different plasma-glass treatments did not change the surface chemistry of the PFOTS-water interfaces.

In contrast to the contact angles, drop charges changed substantially over time. In all treatments, Q-vs-n curves showed the usual high drop charges for the first few drops and then a monotonic decrease to a steady state drop charge. Drop charges directly after preparation were, however, lower than drop charges after two days (Fig. 6d–f). The drop charge further increased after five weeks. This slow recovery of the drop charge was observed for RCA (Fig. 6d) and for Ar plasma-activated glass (Fig. S5a,b).

In comparison to glass activated by high (160 W) and low (40 W) power of O_2_ plasma, at constant pressure (0.3 mbar) and time (35 s), the sliding drop charge on Fl-glass was higher when the glass was activated with lower plasma power. This phenomenon was observed for Ar plasma as well, with similar parameters. Higher plasma power reduced drop charges more, analogous to plasma treatment after silanization (Fig. 5a). On quartz (Fig. 6g), however, 160 and 40 W plasma treatment had a similar effect, confirming the plasma treatment results after fluorination (Fig. 4c). By blowing ionized air over a timescale of hours, the 1st drop charge increased from 0.1 to 0.5 nC (Fig. 6h). Thus, recovery could be accelerated after a few hours of the samples' exposure to the ionizer rather than storing the samples in the air (Fig. 6d–f). One explanation could be the absorption of positive charges from the ionizer into the surface. Similarly, the drop charge increased within the electrode-facing area of the low-pressure plasma chamber (Fig. 2b). However, the recovery of the sliding drop charge after exposing the Fl-glass to the ionizing air blower—as mentioned, the ionizing air blower is an atmospheric plasma discharge in the corona region—took longer than by the electrode-facing treatment of low-pressure plasma (10 h vs. 6 s). One possibility for a longer recovery by ionizer is that plasma discharge in the corona region has relatively low production rates and fluxes of active species^83^.

These results lead to two conclusions. First, drop charging is not only determined by the topmost atoms of the hydrophobic surface, but also by the glass-PFOTS interfacial region. Secondly, the glass-PFOTS interfacial region undergoes an adaptation process over several weeks.

## Conclusion

The phenomenon of drop charging induced by water slide electrification is substantially influenced by plasma treatment. For plasma treatment of low and moderate plasma intensity and duration, charge separation depends on the specific position of a sample in a plasma. When the hydrophobic surface faces the electrode and points towards the sheath region, where the positive space charge exists, charge separation of sliding water drops increases. Drops acquire a more positive charge compared to those without prior plasma treatment. This enhancement in drop charge is attributed to the deposition of positive charges onto the surface, facilitating the adsorption of additional hydroxyl groups upon contact with water, thereby increasing the surface's excess of hydroxyls. As the drop moves, more negative ions remain on the surface, resulting in a net positive charge for the drop.

Conversely, when the hydrophobic surface faces the bulk plasma, the charge of sliding drops diminishes. This results is attributed to two effects: Firstly, plasma-induced alterations in the chemical composition of the top layer render the surface more hydrophilic, reducing the contact angle and making charge transfer less effective^71^. Secondly, superficially deposition of negative charges in the surface. Bulk plasma treatment leads to an increased negative surface charge, which in turn repels hydroxyl ions, diminishing their surface excess and reducing the transfer of hydroxyls to the solid substrate behind the drop. Consequently, fewer positive counter ions remain within the drop. This observation is corroborated by zeta and Kelvin potential measurements. Additionally, XPS data reveal that plasma cleaved C–C bonds partially oxidize the PFOTS coating and reduce the thickness of the coating by etching.

The extent of drop charging depends on the substrate material. On quartz substrates, the drop charges reach saturation significantly faster compared to glass and SU8 substrates. This rapid saturation on quartz is attributed to a long neutralization time, which is most likely due to the high electric resistance of quartz.

Glass, commonly utilized as a substrate in slide electrification experiments, is typically hydrophobized by silanes. To bind the silanes the glass is usually activated chemically or by plasma before hydrophobization. We have demonstrated that the RCA chemical activation, and plasma activation decrease drop charging on hydrophobized surfaces. However, when storing samples in ambient air over several days or weeks, the surface tends to recover, leading to an increase in drop charging by more than a factor of two suggests that by adjusting specific plasma parameters, drop charge can be controlled without affecting contact angles.

## Supplementary Information

Supplementary Information.

## Data Availability

The datasets generated and analyzed in the current study are available from the corresponding author on reasonable request.
