# Supplementary material for: Control of spontaneous charging of sliding water drops by plasma-surface treatment
Source: Sci Rep. 2024 May 9;14:10640. doi: 10.1038/s41598-024-60595-5 (PMC11609279; doi:10.1038/s41598-024-60595-5)
Supplement: Supplementary file 1 — Supplementary Information. [file 41598_2024_60595_MOESM1_ESM.docx]

Control of spontaneous charging of sliding water drops by plasma treatment

Fahimeh Darvish^1^, Sajjad Shumaly^1^, Xiaomei Li^1^, Yun Dong, Diego Diaz^1^, Mohammad Reza Khani^2^, Doris Vollmer^1^, Hans-Jürgen Butt^1^*

^1^ Max Planck Institute for Polymer Research, Ackermannweg 10, D-55128, Mainz, Germany

^2^ Laser and Plasma Research Institute, Shahid Beheshti University, G.C., Evin, 1983963113 Tehran, Iran

KEYWORDS: Water contact electrification, low-pressure plasma treatment, plasma-sheath, adaptation, surface charge neutralization

**Table S1**. Elemental composition and ratios of pristine PFOTS, sheath and plasma treated samples based on XPS survey spectra.

| Sample name | Atomic percentage% | | | | Atomic ratio |
| --- | --- | --- | --- | --- | --- |
|  | O | C | F | Si | O/F |
| Pristine PFOTS | 16.1 | 21.6 | 39.8 | 22.5 | 0.4 |
| Electrode-facing | 17.9 | 20.2 | 36.8 | 25.1 | 0.5 |
| Plasma-facing | 26.7 | 15.8 | 24.9 | 32.7 | 1.8 |

**High resolution of XPS spectra in C1s.** In the C 1s region (284-290 eV) there are three main peaks centered around 286, 292, and 294 eV (Fig 3d-II-IV). The first peak was relatively wide and is believed^1^ to result from the presence of (at least) two unresolved photo emission lines. One originates from the initial carbon atom attached to silicon, and the other from the subsequent carbon atom, which is connected to the first fluorinated carbon (CH_2_-CF_2_), Frechette et al. assigned similarly^66^.

In contrast to the electrode-facing sample, the plasma-facing sample was exposed to a higher density of active species (ions, electrons, photons, free radicals). It led to a decrease in peak areas for CF_2_, CF_3_. In all cases (Fig 3d-II-IV), the CF_3_/CF_2_ ratio was the same (0.23-0.26). Although the peak area of CF_3_ (peak area: no plasma: 2586 electrode-facing: 2430 plasma-facing: 1467 ), and CF_2_ (peak area: no plasma: 9942 electrode-facing: 9798 plasma-facing: 6370) decreased after treatments, the CF_3_/CF_2_ ratio remained almost constant. This constant ratio was observed before^75^. If the plasma-surface reactions involved the F atom abstraction from the (CF_3_)(CF_2_)_5_(CH_2_)_2_-Si adsorbate, one would anticipate different CF_3_/CF_2_ ratios. This indicates that C-C bonds were cleaved by plasma. A possible scenario is that the monolayer thickness decreased from the top to the CF_2_-CH_2_ bond. The peak area of CF_2_-CH_2_ remained relatively unchanged, means plasma affects primarily the chemistry at the topmost of the surface^47,74^. Due to the almost constant C-C/CH_2_ peak area of PFOTS head groups, cleavage of the C bond has a low probability from the Si of the head groups. Furthermore, ellipsometry revealed that the thickness of the PFOTS layer decreased after sheath treatment from 3.0 ± 0.2 nm to 2.6 ± 0.3 nm. The PFOTS layer partially resists sheath etching due to the high strength of the C-F bond (480 KJ mol^-1^), while PS-glass, without C-F bonds, became super hydrophilic with the same sheath treatment parameters (8 W, 6 s, 0.22 mbar).

**
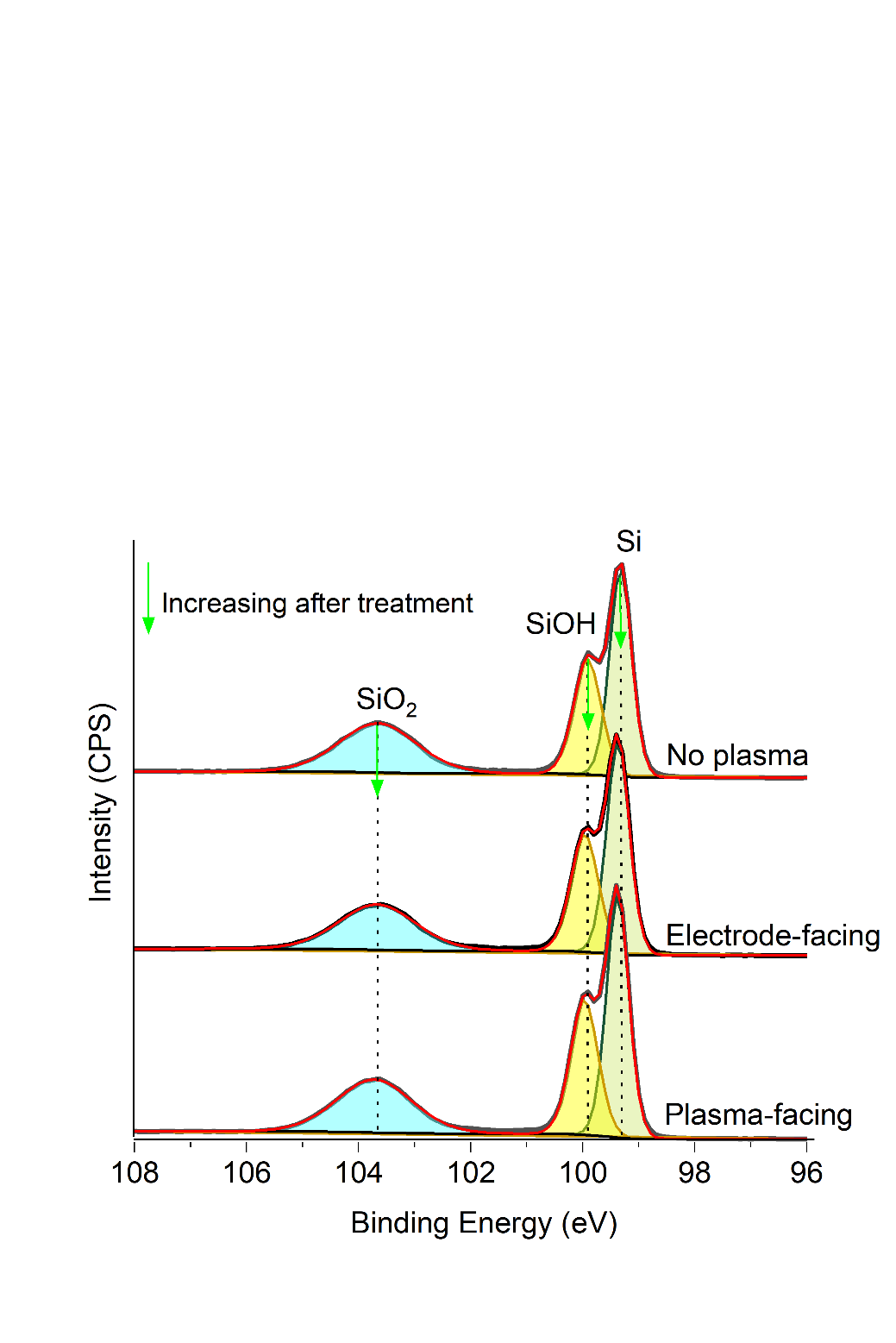
**

**Figure S1**. XPS spectra of Si (2p) recorded on Fl-Si wafers coated with PFOTS. The three spectra show the pristine sample, electrode-facing, and plasma-facing sample exposed to O_2_ bulk plasma. Pressure: 0.22 mbar, time: 6 sec, power: 8 W.


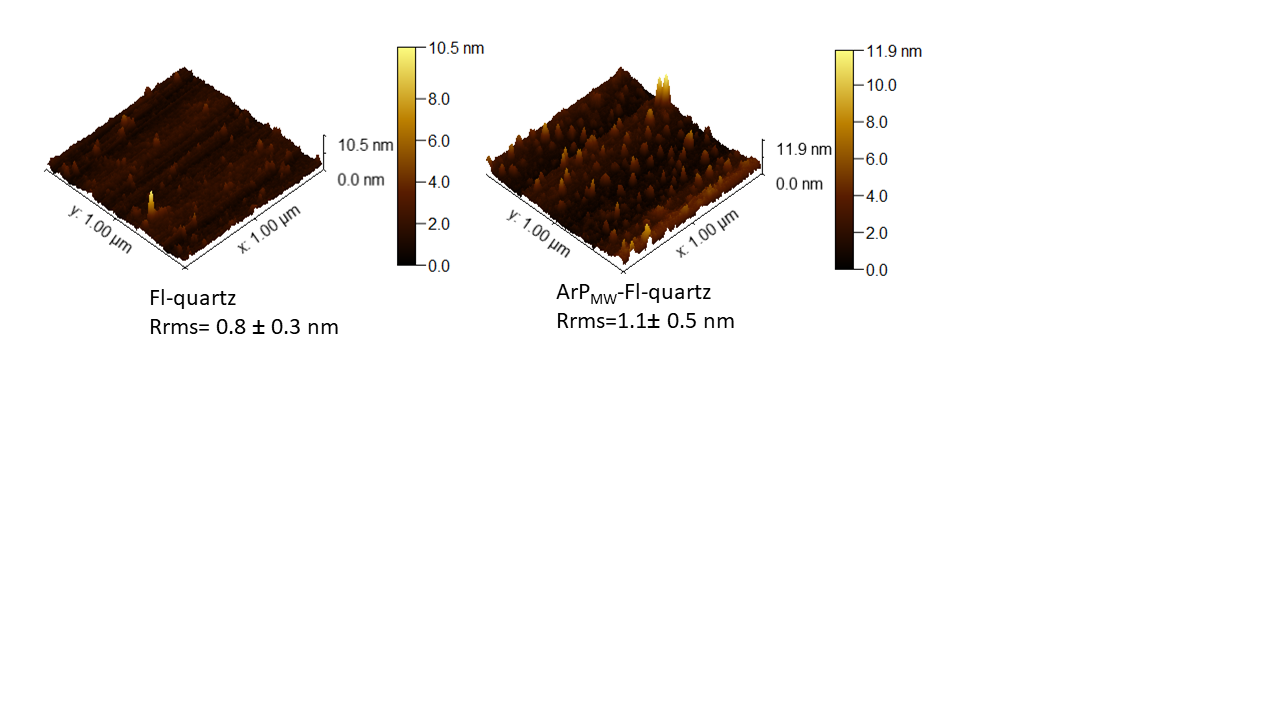


**Figure S2.** AFM images of Fl-quartz before and after 15 s of 15 W Ar plasma treatment. The root mean square roughness was determined over areas of 1 × 1 µm^2^.


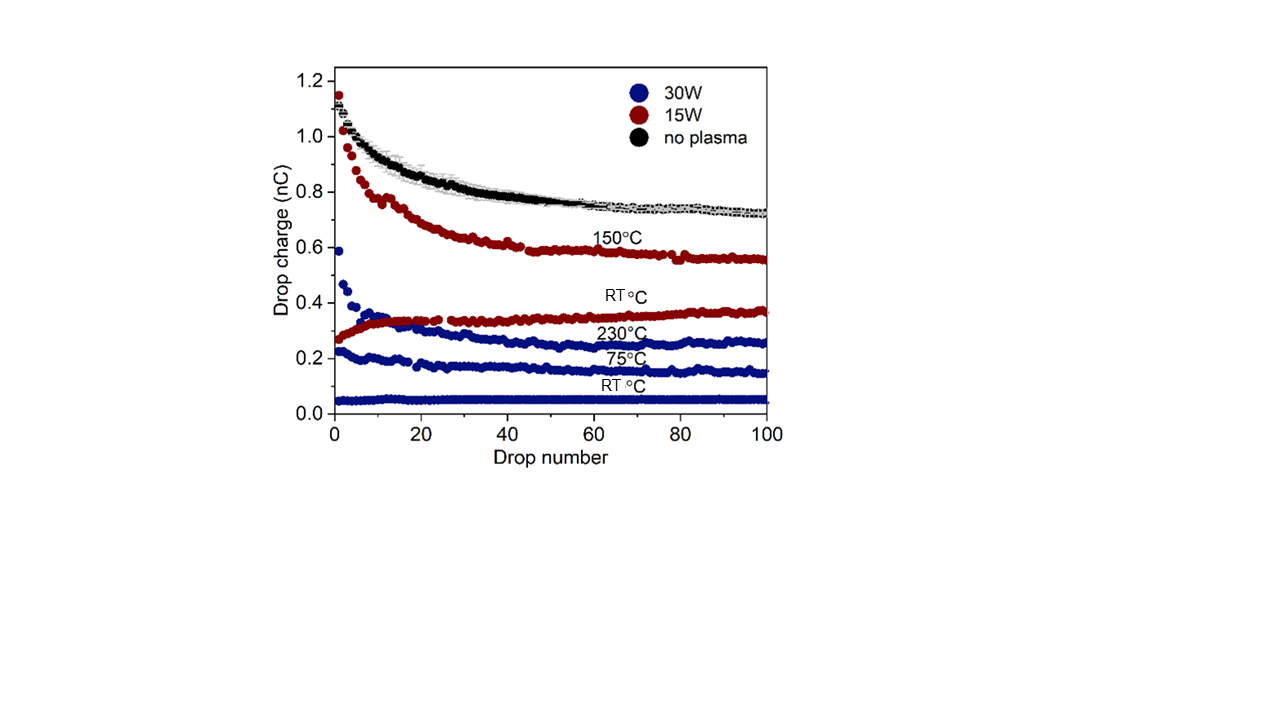

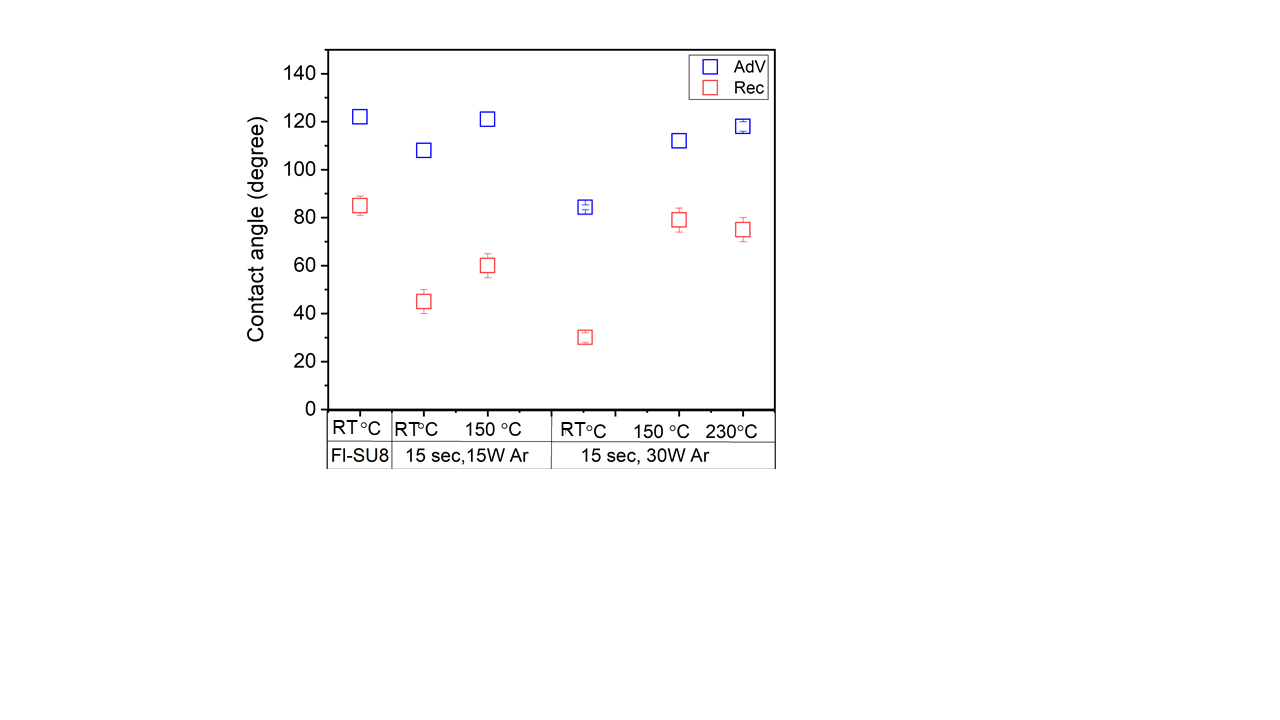


**Figure S3**. Neutralization of plasma treated surfaces after heating. a) The drop charge increased after heating the ArP_MW_-Fl-SU8 samples for 15 W and 30 W for 15 s. Non-heated plasma-treated samples are labeled RT for room temperature. Saturated drop charges increased by 0.2 nC compared to non-heated plasma treated samples, 10 min of heating at 150°C for 15 W and 230°C for 30 W. SU8 samples were fluorinated at high humidity (60-65%). b) advancing and receding contact angle confirm the recovery of hydrophobicity after heating.


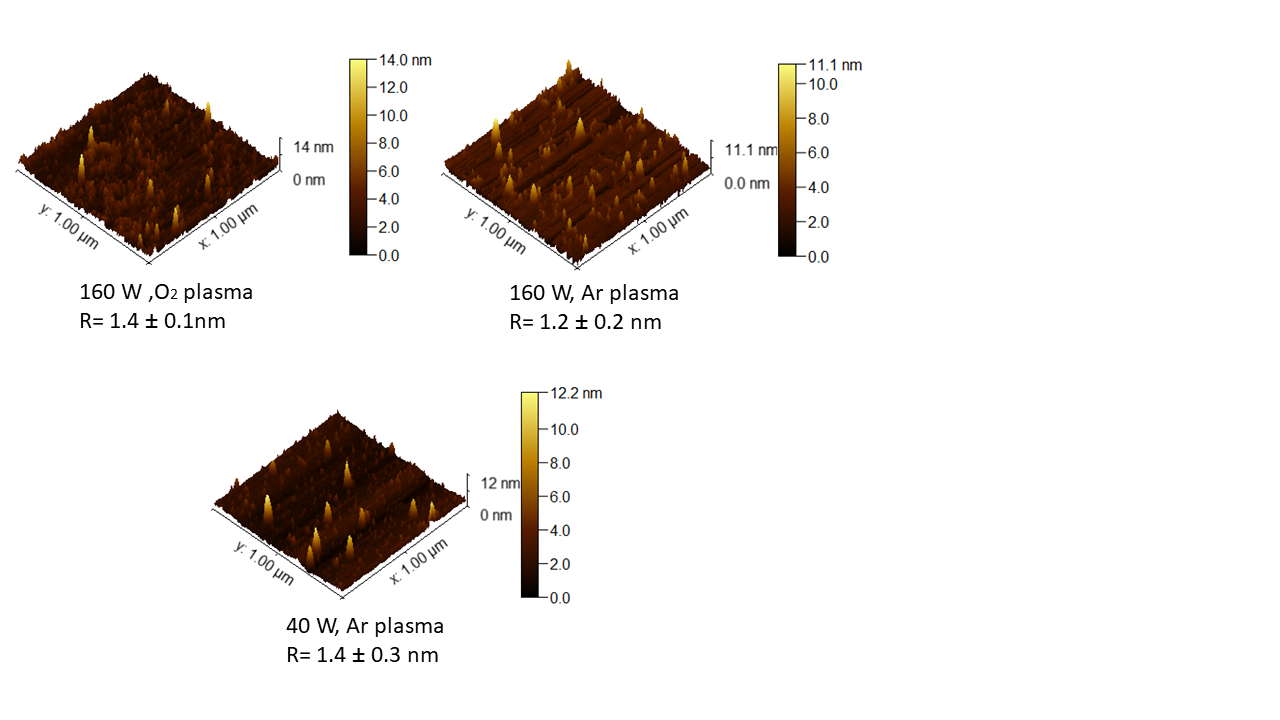


**Figure S4.** RMS surface roughness of Fl-glass was measured after subjecting it to different plasma activation processes. The glasses were activated using Ar and O_2_ plasma in the MW chamber at varying power levels (160 W and 40 W) with constant exposure time (35 s). The root mean square roughness was determined over areas of 1 × 1 µm^2^.


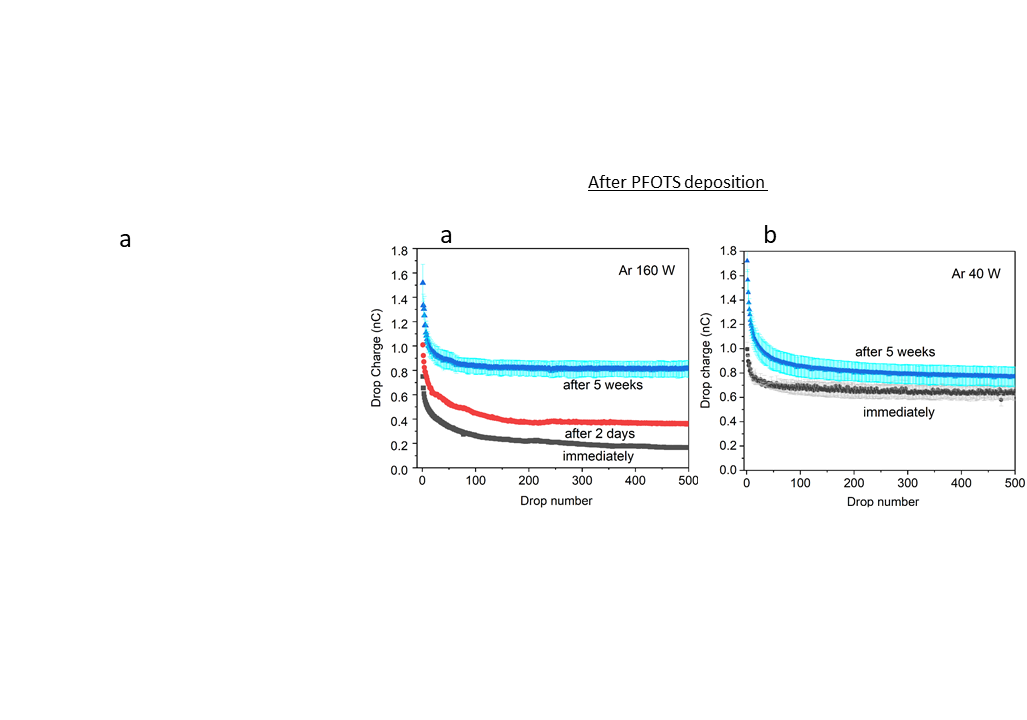


**Figure S5.** Effect of plasma power on the pristine glass and sliding drop charge on Fl-glass. The Drop charge after fluorination of activated glasses by a) 160 W, and b) 40 W Ar plasma within constant time (35s).
